# Effectiveness of Adding Neuromuscular Training to Conventional Physiotherapy in Adults with Patellofemoral Pain Syndrome: A Randomized Controlled Trial

**DOI:** 10.3390/jcm15218668

**Published:** 2026-11-06

**Authors:** Büşra Arısüt, Suzan Aydın, Beyzanur Dikmen Hoşbaş, Ömer Şevgin

**Affiliations:** 1 Department of Physiotherapy and Rehabilitation, Institute of Health Sciences, Üsküdar University, 34662 Istanbul, Türkiye; busraarisutt@gmail.com; 2 Department of Physiotherapy and Rehabilitation, Faculty of Health Sciences, Istanbul Gelişim University, 34310 Istanbul, Türkiye; suaydin@gelisim.edu.tr; 3 Department of Physiotherapy and Rehabilitation, Faculty of Health Sciences, Üsküdar University, 34662 Istanbul, Türkiye; beyzanur.dikmenhosbas@uskudar.edu.tr

**Keywords:** patellofemoral pain syndrome, neuromuscular training, exercise therapy, pain, balance, sleep quality

## Abstract

**Background/Objectives:** Patellofemoral pain syndrome (PFPS) is associated with pain, functional limitation and impaired balance. This study investigated whether adding neuromuscular training to conventional physiotherapy provides additional benefit over conventional physiotherapy alone in adults with PFPS. **Methods:** In this single-blind randomized controlled trial (NCT06407414), 56 adults aged 30–60 years with PFPS were randomized to conventional physiotherapy alone (control) or conventional physiotherapy plus neuromuscular training, each performed three times per week for 12 weeks as a home-based program with physiotherapist follow-up every three weeks. The analysis was per protocol and included the 50 of 56 randomized participants (25 per group) who completed the trial. The primary outcome was the Kujala Patellofemoral Score. Secondary outcomes were pain (Visual Analog Scale, VAS), the 30-Second Chair Stand Test, the Y Balance Test, the International Physical Activity Questionnaire–Short Form (IPAQ) and, as an exploratory outcome, the Pittsburgh Sleep Quality Index (PSQI). **Results:** After treatment, the neuromuscular training group had higher Kujala scores (median 77 vs. 65; r = 0.81, 95% CI 0.69–0.89) and lower VAS scores (r = 0.72, 95% CI 0.55–0.83), and performed better on the chair stand test (mean difference 2.60 repetitions, 95% CI 0.88–4.32) and IPAQ-walking; these differences remained significant after Bonferroni correction for 12 comparisons, although for the chair stand test this was not consistent in a supplementary change-score analysis. Y Balance Test values are reported descriptively only, because the groups differed at baseline and the analytical definition of the reported values (stance limb) could not be verified against the original records. Between-group differences in the other IPAQ domains and the PSQI were not significant and were inconclusive. **Conclusions:** Under the conditions of this trial, adding neuromuscular training to conventional physiotherapy produced greater short-term improvements in pain and function than conventional physiotherapy alone. No conclusion is drawn regarding dynamic balance, and the effect on sleep quality remains uncertain.

## 1. Introduction

Patellofemoral pain syndrome (PFPS) is a prevalent orthopedic disorder that affects adolescents and young adults [1]. PFPS is common, with an annual prevalence of 22.7% reported in the general population and 28.9% in adolescents [2], and a 1.5–3 times higher prevalence in women than in men [3]. The etiology of PFPS is multifactorial, with lower extremity weakness, particularly of the quadriceps, malalignment, foot deformities (e.g., increased subtalar pronation) and tightness of the lateral retinaculum, iliotibial band and hamstring muscles being among the most common contributing factors [4,5]. Although PFPS is most frequently reported in adolescents and young, physically active individuals, it is increasingly recognized as a persistent or recurrent condition in adulthood, and long-standing anterior knee pain is also commonly encountered in adult outpatient physiotherapy settings [4,6].

PFPS is characterized by diffuse anterior, retropatellar (behind the kneecap) or peripatellar (around the kneecap) knee pain. Crepitation in the knee joint is also common and is exacerbated by activities that increase compressive loading of the patellofemoral joint, such as squatting, prolonged sitting, running, and climbing and descending stairs [7,8]. In addition to pain, patients with PFPS exhibit altered muscle activity patterns, particularly in the quadriceps, resulting in patellar malpositioning, which may affect balance and motor control [9]. Several studies have shown that patients with PFPS have deficient postural control during balance testing on an unstable platform and during the Star Excursion Balance Test [10,11].

A decline in functional status may be precipitated by reduced flexibility of the rectus femoris, hamstrings, tensor fasciae latae and iliotibial band, accompanied by an exacerbation of pain [12]. It has been reported that 71–74% of individuals with PFPS modify their physical activity and functional status in response to pain [13], and patients with PFPS have low scores on self-reported functional scales [6]. PFPS has also been associated with reduced health-related quality of life [14,15], although quality of life was not assessed in the present study. Chronic musculoskeletal pain has also been associated with poorer self-reported sleep quality, and pain and sleep are understood to interact bidirectionally [16,17,18]; however, this relationship has rarely been examined specifically in individuals with PFPS, which was a further motivation for including sleep quality as an outcome in the present study.

Conservative treatment of PFPS has focused on trunk and hip muscle strengthening combined with strengthening of the knee musculature [19,20], as reductions in pain and improvements in motor function have been observed with these approaches [21,22]. Exercise programs for PFPS should address multiple regions, including the hip muscles, trunk stability, quadriceps, hamstrings and the iliotibial system [23]. There is no consensus regarding the specific movement patterns present in patients with active patellofemoral pain; however, an inability to eccentrically control hip adduction and internal rotation can lead to more pronounced dynamic lower limb valgus during landing, squatting and running. Proximal mechanisms have been hypothesized to play an important role in neuromuscular control strategies that control and compensate for knee loading during complex movements, and neuromuscular training has been shown to exert a positive influence on hip kinematics and kinetics [24].

Given these kinematic characteristics, integrating neuromuscular training into conventional physiotherapy may be of value. Patients with PFPS demonstrate abnormalities in lower limb mechanics and dynamic control [25]. However, completed randomized trials testing whether neuromuscular or motor control training adds benefit to exercise-based treatment have reported conflicting results. Motealleh et al. found that four weeks of core neuromuscular training added to routine physiotherapy improved pain, balance and functional performance more than routine physiotherapy alone [26], whereas Silva et al., who added neuromuscular training from the fourth week of a 12-week strengthening program, and Rabelo et al., who added motor control training to muscle strengthening, observed no substantial additional improvement in clinical or kinematic outcomes [27,28,29]. Because these trials enrolled only women, the incremental value of neuromuscular training for adults of both sexes presenting with long-standing anterior knee pain to outpatient physiotherapy remains unclear. The present study therefore addressed a specific question: whether a neuromuscular training program combining core stability, balance, strength and plyometric components, introduced from the start of treatment and progressed over 12 weeks, provides additional benefit over a conventional physiotherapy program alone in adults aged 30–60 years with PFPS. We hypothesized that, compared with conventional physiotherapy alone, the combined program would produce greater improvements in the primary outcome (function, assessed with the Kujala score) and in pain, functional performance, dynamic balance and physical activity. Sleep quality was examined as an exploratory outcome without a directional hypothesis, because the bidirectional relationship between pain and sleep does not by itself indicate that neuromuscular training should improve sleep more than conventional exercise.

## 2. Materials and Methods

### 2.1. Study Design

This prospective, single-blind, parallel-group randomized controlled trial was conducted between May and September 2024 at Fatih No. 8 Family Health Center. The first participant was enrolled on 20 May 2024. Ethical approval was obtained from the Non-Interventional Research Ethics Committee of Üsküdar University on 26 February 2024 (meeting no. 02; approval no. 61351342/February 2024-05), before participant recruitment began. The study was conducted in accordance with the Declaration of Helsinki, all participants provided written informed consent, and the trial was registered at ClinicalTrials.gov on 6 May 2024 (NCT06407414). This report follows the CONSORT 2025 statement for parallel-group randomized trials.

### 2.2. Sample Size

**Figure 1:**
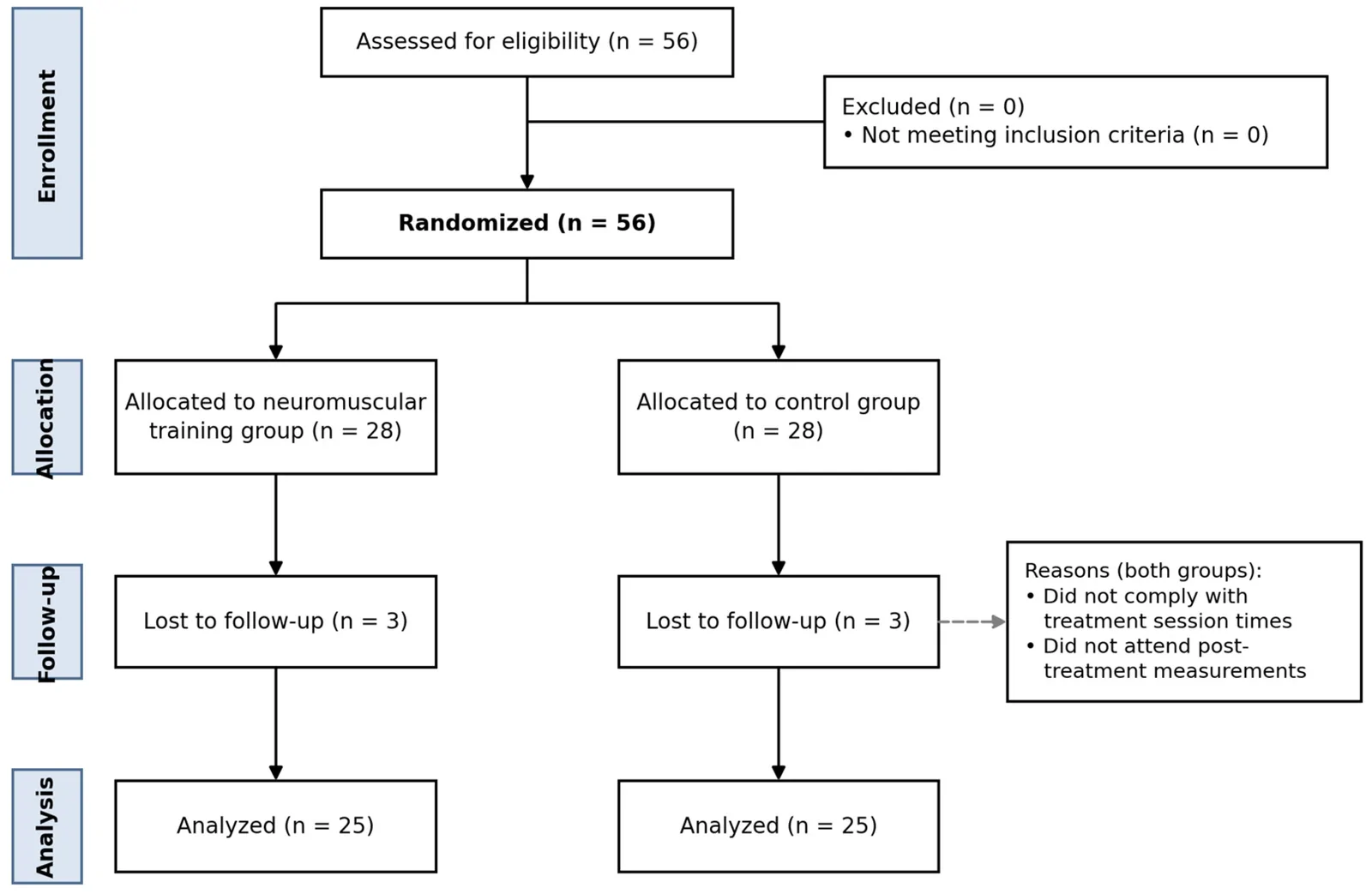
CONSORT flow diagram of the participants. The dashed arrow points to the reasons for loss to follow-up, which were the same in both groups.

Sample size was estimated a priori using G*Power (version 3.1; Heinrich-Heine-Universität Düsseldorf, Düsseldorf, Germany) [30], with the Kujala Patellofemoral Score, the study’s primary outcome, as the basis for the calculation. The effect size used for planning (d = 1.04) was derived from Motealleh et al. [26], the most directly comparable trial available at the design stage, which evaluated a structurally similar intervention (core neuromuscular training added to routine physiotherapy versus routine physiotherapy alone) in women with PFPS. That trial reported between-group effect sizes of d = 1.44 for improvement in pain (VAS) and d = 1.67 for improvement in the Kujala score, calculated from the reported group means and standard deviations. A more conservative value of d = 1.04 was adopted to avoid underestimating the required sample size; this deliberately increased, rather than reduced, the required sample relative to a calculation based on the published estimates. It is nonetheless acknowledged that d = 1.04 still exceeds Cohen’s conventional threshold for a large effect (d = 0.80) [31], that a smaller assumed effect would have required a correspondingly larger sample, and that this is a limitation of the design. For a two-tailed independent-samples *t*-test with α = 0.05 and a statistical power of 0.95, the calculation yielded a minimum of 26 participants per group (52 in total); 28 participants per group (56 in total) were recruited to allow for possible losses to follow-up. Of the 56 randomized participants, 3 in each group were lost to follow-up (Figure 1), leaving 50 participants (25 per group) for analysis. No post hoc (observed) power calculation was performed on the achieved sample, for the reasons given in Section 2.7 [32]; the implications of the achieved sample size are instead conveyed by the effect sizes and 95% confidence intervals reported for every between-group comparison (Section 3).

### 2.3. Randomization and Blinding

After completion of the baseline assessment, participants were allocated in a 1:1 ratio to the neuromuscular training group or the control group using simple unrestricted computer-generated randomization (Research Randomizer, https://www.randomizer.org/) without blocking or stratification. The allocation sequence was generated before enrolment by an independent researcher who had no role in recruitment, baseline assessment, treatment delivery, outcome assessment or statistical analysis. Group assignments were placed in sequentially numbered, opaque, sealed envelopes prepared according to the generated sequence, and the envelopes were opened consecutively only after a participant had been enrolled and the baseline assessment completed. After the study, the original randomization sequence and the allocation log were independently cross-checked by a researcher who had not participated in recruitment, treatment delivery, outcome assessment or statistical analysis. Outcome assessments were performed by an assessor blinded to group allocation; participants and the treating physiotherapist could not be blinded because of the nature of the exercise-based intervention.

### 2.4. Participants

Informed consent was obtained from 56 participants whose eligibility was confirmed by an orthopedic physician. Participant flow is shown in Figure 1. Six participants (3 per group) were lost to follow-up after randomization; the recorded reasons were non-compliance with scheduled treatment session times and non-attendance at post-treatment measurements.

Inclusion criteria were age between 30 and 60 years; anterior or retropatellar knee pain with a Visual Analog Scale (VAS) score of at least 3 for a minimum of three months [27]; pain provoked by at least two of the following: running, walking, jumping, climbing and/or descending stairs, prolonged sitting or kneeling, squatting, isometric knee extension at 60° of flexion, and palpation of the medial or lateral patellar facet [20]; and access to a computer, tablet or phone with an internet connection. Although PFPS is classically described as most prevalent among adolescents and young, physically active individuals, the 30–60-year age range was selected to reflect the demographic profile of adults presenting with chronic anterior knee pain to the outpatient clinic in which recruitment took place, and to examine neuromuscular training in an adult population in whom it has been less extensively studied; this is acknowledged as a limitation with respect to generalizability (Section Strengths and Limitations). Exclusion criteria were a history or clinical signs of patellofemoral dislocation, subluxation or severe osteoarthritis; previous knee or other lower extremity surgery; clinical evidence of knee ligament, bursa, meniscus or synovial plica involvement or other specific knee pathology; lower extremity tendinopathy; low back pain; sacroiliac joint pain; pregnancy; and any neurological, cardiovascular or rheumatological condition, or any other condition compromising understanding of the procedures or affecting physiotherapy testing and treatment [27].

### 2.5. Interventions

Participants received detailed information about all study procedures before providing written informed consent. Exercise was performed three times per week for 12 weeks (36 sessions). The program was taught under the supervision of a physiotherapist at the start of each three-week period, documented in a brochure and then performed as an individual home program. Weekly reminder messages were sent to participants. The physiotherapist conducted follow-up visits at three-week intervals to assess adherence and to progress the program: at each visit, exercise difficulty was advanced for patients who demonstrated correct technique and adequate tolerance, primarily through therapist-judged increases in range of motion/depth, movement speed and control demands within the same exercise repertoire, while sets and repetitions were kept constant throughout the 12 weeks (Table 1). Exercise intensity was not quantified with a standardized scale; it was adjusted by the physiotherapist, who made the final decision at each follow-up visit, taking into account the participant’s pain response and movement quality. Patients who had not yet mastered correct technique or who lacked adequate tolerance continued at the previous level until the next visit. Adherence was monitored by the physiotherapist both online, through the weekly messages, and face to face at the follow-up visits. Participants recorded every prescribed session on an exercise monitoring chart, which the physiotherapist reviewed and verified against participant report at each follow-up visit. Adherence was expressed as the number of completed sessions divided by the number of prescribed sessions (36), and attendance at the three-weekly follow-up visits was recorded separately. Participants in both groups were asked not to receive any other treatment for their knee pain during the 12-week intervention period, and this was checked by the physiotherapist at each follow-up visit.

*Control group*. Participants in the control group performed a conventional physiotherapy program comprising a warm-up with general mobility exercises, strengthening exercises, stretching exercises and a cool-down, three days per week for 12 weeks, without neuromuscular training. After demonstration and confirmation of the patient’s understanding, the exercises were prescribed as a home program. Control participants were taught, monitored and followed up on the same schedule as the neuromuscular training group (three-weekly visits, weekly reminder messages and exercise monitoring charts). The conventional program comprised walking as a warm-up, active knee and hip mobility exercises, six strengthening exercises targeting the quadriceps and hip muscles (3 × 10–12 repetitions each), stretching of the quadriceps, hamstring and gastrocnemius muscles (2 × 30 s per muscle and side) and slow walking as a cool-down; the exercises and dosage are listed in Table 1a. The same conventional program was performed by the neuromuscular training group.

**Figure 2:**
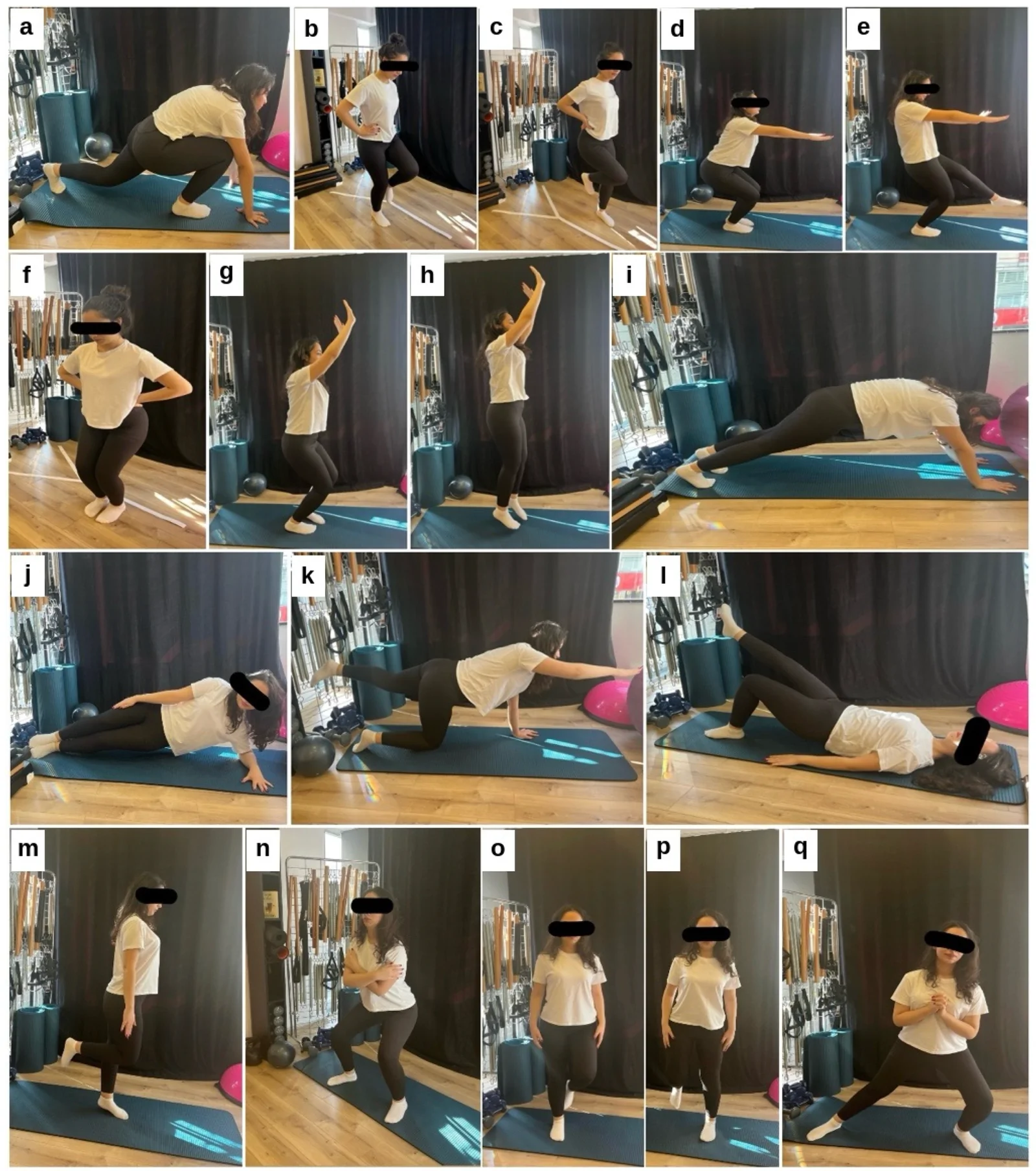
Neuromuscular training program: lunge to hamstring stretch (**a**), side jumps (**b**,**c**), squat (**d**), single-leg squat (**e**), forward–backward hops (**f**), double-leg vertical jump (**g**,**h**), plank (**i**), side plank (**j**), bird dog (**k**), single-leg glute bridge (**l**), single-leg stand (**m**), monster walk (**n**), lateral jumps (**o**,**p**) and lateral lunge (**q**). Single toe raises (Table 1) are not illustrated.

*Neuromuscular training group*. Participants in this group received neuromuscular exercise training delivered by a physiotherapist in addition to the conventional physiotherapy program (Figure 2). After the patient’s correct performance had been verified, the neuromuscular training program was performed as a home program three days per week for 12 weeks. The neuromuscular training segment lasted 25–30 min per session and was performed in addition to the conventional program (Table 1), so the neuromuscular training group received a greater total exercise dose than the control group; total session durations were not recorded individually. All exercises were performed using body weight alone and required no additional equipment. The program was adapted for patients with PFPS and included exercises targeting core stability, balance, strength and plyometric ability. The exercises, dosage and progression approach are detailed in Table 1b.

### 2.6. Outcome Measures

**Figure 3:**
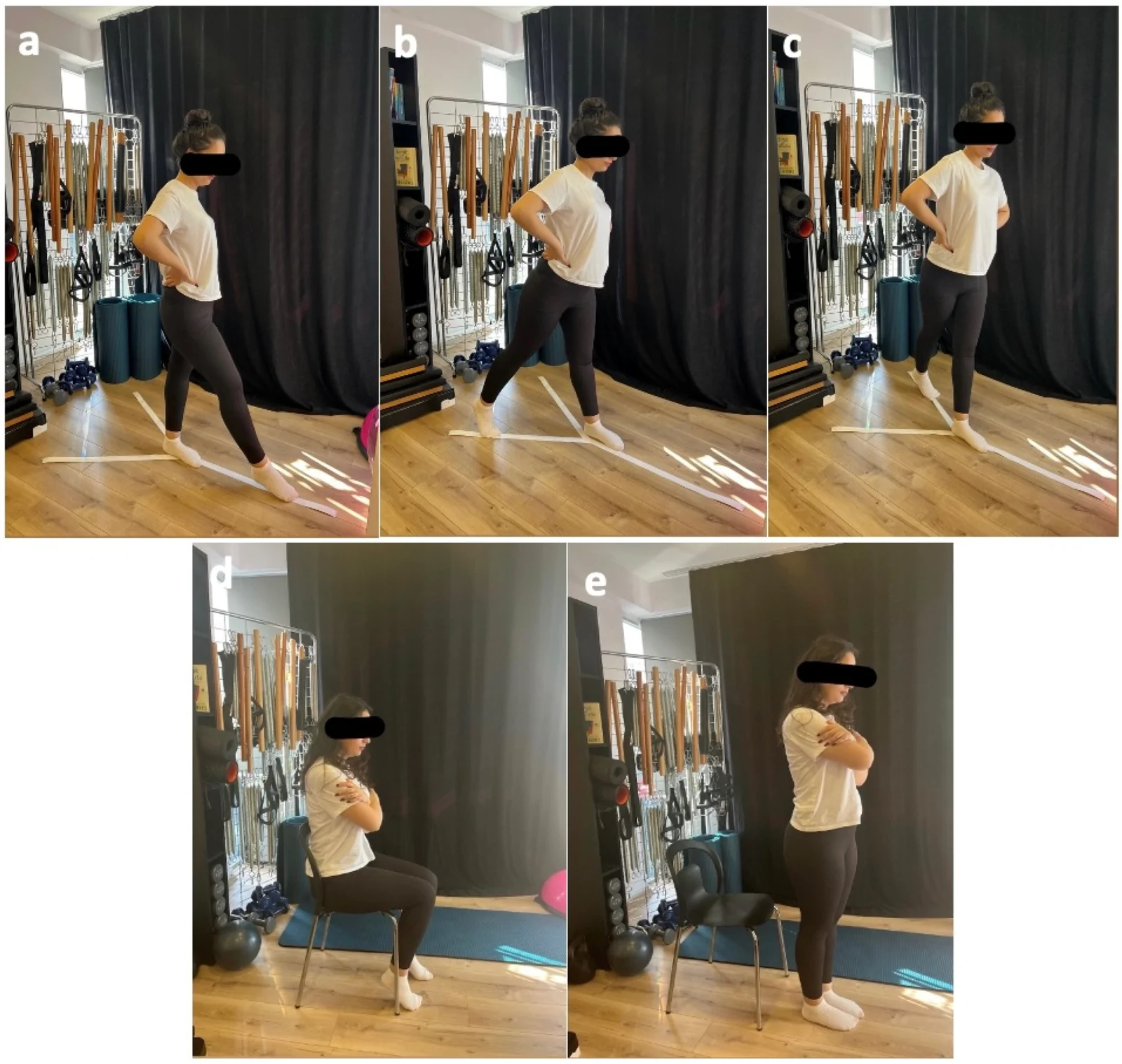
Y Balance Test anterior (**a**), posteromedial (**b**) and posterolateral (**c**) reach; 30-Second Chair Stand Test (**d**,**e**).

Sociodemographic data (age, sex, body mass index, education level, occupation, dominant side and side of the painful knee) were recorded. Pain was assessed using the VAS; function using the Kujala Patellofemoral Score and the 30-Second Chair Stand Test; balance using the Y Balance Test (YBT) (Figure 3); physical activity using the International Physical Activity Questionnaire–Short Form (IPAQ-SF); and sleep quality, as a secondary exploratory outcome, using the Pittsburgh Sleep Quality Index (PSQI). All assessments were performed at baseline and after the 12-week program.

#### 2.6.1. Visual Analog Scale

The VAS measures pain intensity on a continuous scale from 0 (no pain) to 10 (worst imaginable pain) and was used to assess the severity of knee pain. Participants marked a point on a 10 cm horizontal line anchored by “no pain” and “unbearable pain”; the distance was measured with a ruler and recorded in centimeters [33].

#### 2.6.2. Kujala Patellofemoral Score

The Kujala Patellofemoral Score, developed by Kujala et al. in 1993 for the functional evaluation of knee problems of patellofemoral origin, assesses pain, subluxation and swelling during activities such as running, stair climbing, jumping, squatting and prolonged sitting with the knees flexed, as well as the need for walking aids, limitation of knee flexion and quadriceps atrophy [34]. Scores range from 0 (severe pain and dysfunction) to 100 (normal knee without pain or functional impairment). The Kujala score was the study’s primary outcome (Section 2.2). The validity and reliability of the Turkish version were established by Kuru et al. [35].

#### 2.6.3. 30-Second Chair Stand Test

This test evaluates sit-to-stand performance, lower limb strength and dynamic balance. A chair without armrests, with a straight backrest and a seat height of 43.18 cm (17 inches), was used. Participants sat with the back straight, feet flat on the floor and arms crossed over the chest, and on the command “start” performed as many full stands as possible in 30 s; the number of full stands was recorded as the score. Two to three familiarization trials preceded the test trial [36].

#### 2.6.4. Y Balance Test

The YBT is a simple and reliable measure of dynamic balance performed in single-leg stance, requiring strength, flexibility, core control and proprioception. While maintaining balance on one foot, the participant reaches with the contralateral foot in the anterior, posteromedial and posterolateral directions [37]. In the present study, the maximal reach distance in each direction was recorded in centimeters and analyzed separately. The test was performed on both limbs. Three trials were performed in each reach direction, and the highest reach distance was retained for analysis. The original paper assessment forms were securely destroyed after the data had been transferred to electronic format, to protect participant confidentiality, and no contemporaneous documentation specifying the stance limb or the selection rule used for the reported values (e.g., symptomatic limb, contralateral limb or an average) could be identified; it therefore cannot be confirmed that the same limb and selection rule were applied at baseline and follow-up. For this reason, the YBT results are reported descriptively for completeness and are not used to support the conclusions of this study. Limb length was not recorded; reach distances were therefore analyzed in centimeters without normalization to limb length.

#### 2.6.5. International Physical Activity Questionnaire–Short Form

The IPAQ-SF comprises seven questions on walking, time spent in moderate and vigorous physical activity, and sitting time. Energy expenditure was expressed in MET-min/week using standard MET values (walking, 3.3 METs; moderate activity, 4.0 METs; vigorous activity, 8.0 METs), calculated as MET value × minutes per day × days per week; for example, walking for 20 min on four days per week yields 3.3 × 20 × 4 = 264 MET-min/week. Sitting time was recorded in hours per day. Participants were classified as inactive, minimally active or very active [38].

#### 2.6.6. Pittsburgh Sleep Quality Index

The PSQI, developed by Buysse et al., is a self-administered questionnaire that yields seven component scores (subjective sleep quality, sleep latency, sleep duration, habitual sleep efficiency, sleep disturbances, use of sleep medication and daytime dysfunction), each scored 0–3, and a global score of 0–21, with higher scores indicating poorer sleep quality; a global score greater than 5 discriminates poor from good sleepers with high sensitivity and specificity [39]. The PSQI was analyzed as a secondary, exploratory outcome, given the more limited a priori evidence linking neuromuscular training specifically to sleep quality.

### 2.7. Statistical Analysis

Analyses were performed using IBM SPSS Statistics 21.0 (IBM Corp., Armonk, NY, USA). Quantitative variables are presented as mean ± standard deviation or median (minimum–maximum), and qualitative variables as frequency and percentage. Normality was assessed using the Shapiro–Wilk test and box plots. Qualitative variables were compared using the chi-square test. Depending on the distribution, the independent-samples *t*-test or the Mann–Whitney U test was used for between-group comparisons, and the paired-samples *t*-test or the Wilcoxon signed-rank test for within-group comparisons. Effect sizes were calculated for all between-group comparisons: Cohen’s d (with 95% confidence intervals, CIs) for parametric comparisons and the rank-based correlation coefficient r (r = Z/√N) for non-parametric comparisons, with r ≈ 0.10, 0.30 and 0.50 and d ≈ 0.20, 0.50 and 0.80 interpreted as small, medium and large effects, respectively [31]. For normally distributed variables, the mean difference and its 95% CI were also calculated; CIs for r were obtained using the Fisher z transformation. No post hoc (observed) power analysis was performed, because such analyses are a monotone transformation of the *p*-value and cannot inform the interpretation of an observed result [32]. The precision of each estimate is instead expressed through its CI, and non-significant between-group comparisons are interpreted as inconclusive rather than as evidence of the absence of an effect; establishing the latter would require formal equivalence testing against a pre-specified margin in a trial designed for that purpose [40]. The Kujala score was the single primary outcome; VAS, the 30-Second Chair Stand Test, the YBT and the IPAQ were secondary outcomes, and the PSQI was an exploratory outcome. To account for multiplicity, a Bonferroni correction was additionally applied across all 12 post-treatment between-group comparisons reported in Section 3 (adjusted α = 0.05/12 ≈ 0.0042). The primary analysis consisted of the post-treatment between-group comparisons described above. A verified participant-level dataset was not available at the revision stage (see Data Availability Statement), so baseline-adjusted analyses (ANCOVA) could not be performed. As a supplementary analysis only, the between-group difference in mean change from baseline (with its 95% CI) was calculated from the group summary statistics for the normally distributed outcomes (30-Second Chair Stand Test and the posteromedial and posterolateral YBT directions). This supplementary analysis is not equivalent to an individual-level baseline-adjusted analysis, and its CIs and *p* values depend on the assumed baseline–follow-up correlation. Because the correlation between baseline and post-treatment values was unknown, the variance of change was calculated conservatively assuming a correlation of zero, which overestimates this variance when the true correlation is positive; a correlation of 0.5 was examined as a sensitivity assumption. All analyses were performed per protocol (completers only): the six participants lost to follow-up provided no post-treatment data, and no intention-to-treat analysis with imputation was undertaken. The baseline data of the six participants lost to follow-up were deleted after data collection had been completed, because they were not to be included in the per-protocol analysis; their characteristics therefore could not be compared with those of completers, and multiple imputation or other model-based intention-to-treat analyses were not possible. For the same reason, sensitivity analyses imputing the missing outcomes could not be performed. The nominal significance level was *p* < 0.05.

## 3. Results

### 3.1. Participant Characteristics and Adherence

Age, body mass index, sex, education level, dominant side and side of the painful knee did not differ significantly between the groups (*p* > 0.05; Table 2). Adherence data refer to the 50 participants who completed the trial: each of them performed all 36 prescribed sessions, as documented in the exercise monitoring charts, and attended every three-weekly follow-up visit. The six randomized participants (three per group) who did not exercise regularly were lost to follow-up and did not attend the post-treatment assessment (Figure 1); because their participation records were not retained, adherence in the full randomized sample (*n* = 56) cannot be quantified, and the 100% figure should not be interpreted as adherence to the intervention as allocated. Baseline data of these six participants were deleted after data collection was completed, so their characteristics could not be compared with those of the completers. Adverse events were ascertained by the physiotherapist, who asked participants about pain exacerbation, injuries or other complaints related to the exercises at each three-weekly follow-up visit; participants lost to follow-up were not contacted. Among the participants who completed the trial, no adverse events or exercise-related injuries were reported in either group.

### 3.2. Primary Outcome

The Kujala Patellofemoral Score improved significantly from baseline to 12 weeks within both groups (*p* < 0.001 in both groups; Table 3). After treatment, the neuromuscular training group scored significantly higher than the control group (median 77, range 74–84, versus median 65, range 61–78; *p* < 0.001), with a large effect size (r = 0.81, 95% CI 0.69 to 0.89), and this difference remained significant after Bonferroni correction. The group median increased by 22 points in the neuromuscular training group and by 10 points in the control group. Because these are changes in group medians rather than individual change scores, they cannot be compared directly with the 10-point minimal clinically important difference (MCID) reported for this scale [41] and do not establish that the additional benefit is clinically important. Because individual change scores were not available, the proportion of participants achieving the MCID in each group could not be determined.

### 3.3. Secondary Outcomes

After treatment (Table 3), significant between-group differences in favor of the neuromuscular training group were found for VAS (r = 0.72, 95% CI 0.55 to 0.83; large effect) and IPAQ-walking (r = 0.48, 95% CI 0.23 to 0.67; moderate-to-large effect), both of which remained significant after Bonferroni correction. No significant between-group differences were found for IPAQ vigorous activity (r = 0.05, 95% CI −0.23 to 0.32), moderate activity (r = 0.07, 95% CI −0.21 to 0.34), sitting (r = 0.27, 95% CI −0.01 to 0.51) or total activity (r = 0.23, 95% CI −0.05 to 0.48) (*p* > 0.05). The CIs for these four comparisons include both negligible and moderate effects and therefore do not exclude a clinically relevant between-group difference; these results are accordingly interpreted as inconclusive rather than as demonstrating the absence of an effect. Within-group changes from baseline were significant in both groups for all of these outcomes (Table 3).

**Figure 4:**
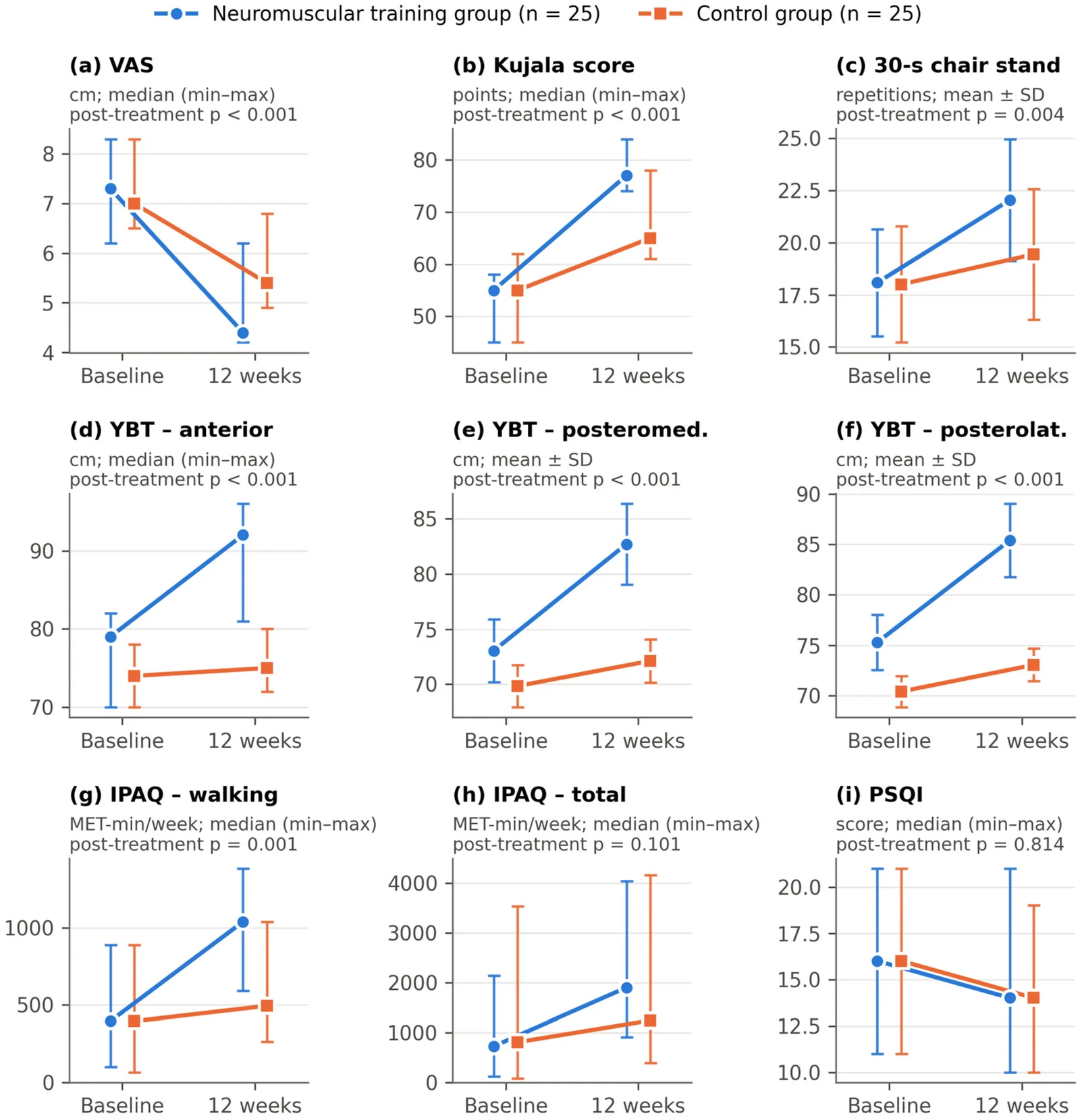
Baseline and 12-week values of the main outcomes in the neuromuscular training and control groups: (**a**) Visual Analog Scale (VAS); (**b**) Kujala Patellofemoral Score; (**c**) 30-Second Chair Stand Test; (**d**–**f**) Y Balance Test (YBT) anterior, posteromedial and posterolateral reach; (**g**) International Physical Activity Questionnaire (IPAQ) walking; (**h**) IPAQ total; (**i**) Pittsburgh Sleep Quality Index (PSQI). Markers show medians with minimum–maximum ranges for non-normally distributed variables and means ± standard deviation (SD) for normally distributed variables, as indicated in each panel; *p* values refer to the post-treatment between-group comparisons reported in Table 3 and Table 4. Group markers are offset horizontally for readability. The IPAQ vigorous, moderate and sitting domains are reported in Table 3. YBT values (**d**–**f**) are shown descriptively; their analytical definition could not be verified (Section 2.6.4).

**Figure 5:**
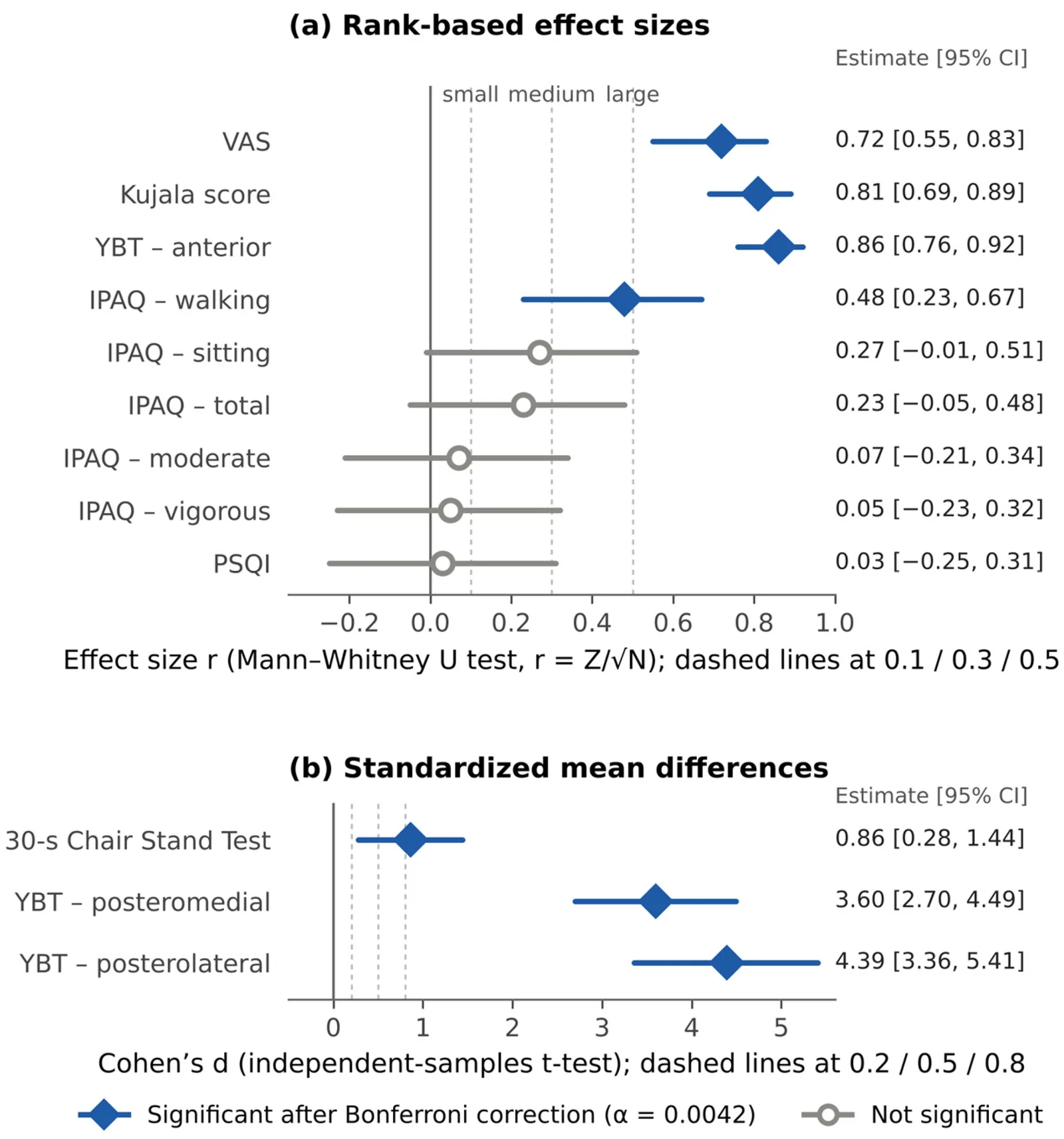
Post-treatment between-group effect sizes with 95% confidence intervals (CIs): (**a**) rank-based effect size r for outcomes compared with the Mann–Whitney U test; (**b**) Cohen’s d for outcomes compared with the independent-samples *t*-test. Filled diamonds indicate comparisons that remained significant after Bonferroni correction (adjusted α = 0.0042); open circles indicate non-significant comparisons. Values of r are unsigned magnitudes; all significant differences favored the neuromuscular training group. The direction of the non-significant rank-based comparisons could not be recovered because a verified participant-level dataset was unavailable. YBT effect sizes are shown descriptively and are not interpreted as treatment effects (Section 2.6.4). Dashed lines mark the conventional thresholds for small, medium and large effects. VAS, Visual Analog Scale; YBT, Y Balance Test; IPAQ, International Physical Activity Questionnaire; PSQI, Pittsburgh Sleep Quality Index.

Results for the 30-Second Chair Stand Test, YBT and PSQI are presented in Table 4. After treatment, significant between-group differences in favor of the neuromuscular training group were found for the 30-Second Chair Stand Test (mean difference 2.60 repetitions, 95% CI 0.88 to 4.32; d = 0.86, 95% CI 0.28 to 1.44) and all three YBT directions (posterolateral: mean difference 12.36 cm, 95% CI 10.74 to 13.98, d = 4.39, 95% CI 3.36 to 5.41; posteromedial: mean difference 10.56 cm, 95% CI 8.88 to 12.24, d = 3.60, 95% CI 2.70 to 4.49; anterior: r = 0.86, 95% CI 0.76 to 0.92) (*p* < 0.05); these differences remained significant after Bonferroni correction. Because the analytical definition of the reported YBT values could not be verified (Section 2.6.4) and the groups differed at baseline, these YBT results are presented descriptively and are not interpreted as evidence of a treatment effect. Because the neuromuscular training group already had higher YBT values at baseline in all three directions, these unadjusted post-treatment differences overestimate the treatment effect. In the supplementary summary-statistic analysis, which is not equivalent to a baseline-adjusted analysis of individual data, the neuromuscular training group improved by 7.48 cm more than the control group in the posterolateral direction (95% CI 5.44 to 9.52; *p* < 0.001) and by 7.36 cm more in the posteromedial direction (95% CI 5.19 to 9.53; *p* < 0.001), compared with unadjusted post-treatment differences of 12.36 and 10.56 cm, respectively. In the anterior direction, the group median increased by 13 cm in the neuromuscular training group and by 1 cm in the control group. For the 30-Second Chair Stand Test, the difference in mean change was 2.52 repetitions (95% CI 0.23 to 4.81; *p* = 0.032) under the conservative assumption and 2.52 repetitions (95% CI 0.89 to 4.15; *p* = 0.003) assuming a correlation of 0.5; this difference therefore did not consistently meet the Bonferroni-adjusted threshold, and its statistical robustness depends on the assumed baseline–follow-up correlation. Given the small post-treatment standard deviations for the YBT, particularly in the control group (1.62–1.96 cm), and the baseline difference between the groups in all three reach directions, the very large Cohen’s d values for this outcome should be regarded as imprecise estimates of the true magnitude of the treatment effect (Section 4). No significant between-group difference was observed in the PSQI score (r = 0.03, 95% CI −0.25 to 0.31; *p* > 0.05); this CI spans effects in both directions and is compatible with a small benefit of either intervention as well as with no difference. Within-group changes from baseline were significant in both groups for all of these outcomes (Table 4). Baseline and post-treatment values of the main outcomes in both groups are illustrated in Figure 4, which also shows the baseline differences between groups in the YBT, and the post-treatment between-group effect sizes with their 95% CIs are summarized in Figure 5.

## 4. Discussion

This study examined the effect of adding a neuromuscular training program to conventional physiotherapy on pain, function, physical activity, balance and sleep quality in patients with PFPS. Both groups improved in pain, function, physical activity, balance and sleep quality after treatment. In the between-group comparison, the neuromuscular training group showed significantly greater improvements in pain, function and physical activity (walking) than the control group, whereas no significant difference was found for sleep quality. Balance (YBT) results are not interpreted as evidence of a treatment effect, because the groups differed at baseline and the analytical definition of the reported values could not be verified.

Strengthening of the core, hip and knee muscles is the recommended approach, as it yields superior improvements in pain and function compared with strengthening of these muscles in isolation [42]. Neuromuscular training focuses on motor control and includes exercises that demand greater strength, coordination and balance [43]. Dynamic and challenging exercises appear necessary to facilitate motor adaptation and to stimulate proprioceptive input; consequently, a neuromuscular training protocol emphasizing sensorimotor control may enhance motor function, which in turn may further improve pain and function [28]. A recent study concluded that a four-week program combining core neuromuscular training with routine physiotherapy was more effective than routine physiotherapy alone in improving pain, balance and functional performance in individuals with PFPS [26]. In the present study, adding a 12-week neuromuscular training program to conventional physiotherapy produced a significantly greater reduction in pain and greater improvements in function and balance than conventional physiotherapy alone.

Not all trials evaluating the addition of neuromuscular or motor control training to a strengthening program in PFPS have reported additional benefit: Silva et al. found that adding neuromuscular training to a strengthening program did not produce additional improvement in clinical or kinematic outcomes in women with PFPS [28], and a related trial similarly reported no substantial additional benefit of motor control training over muscle strengthening alone [29]. Several methodological differences may help explain why the present findings diverge from these reports. First, although the trial by Silva et al. also lasted 12 weeks, neuromuscular training was introduced only from the fourth week, after an initial strengthening phase [27,28]; in the present study, neuromuscular training was performed from the first week, so participants received a longer exposure to this component. Second, the present study included both men and women, whereas the earlier trials [28,29] enrolled only women; however, whether sex influences the response to neuromuscular training cannot be determined from the present data, because the study was neither designed nor powered for subgroup analyses. Third, the neuromuscular training protocol used here combined core stabilization, balance, strength and plyometric components delivered progressively over 12 weeks (Table 1), which differs in content and dosage from the protocols evaluated previously; in addition, because the neuromuscular program was added to the conventional program, the total exercise dose was greater in the experimental group than in the control group. Finally, the outcome measures differed: the present study relied on patient-reported and performance-based clinical measures (VAS, Kujala score, 30-Second Chair Stand Test, YBT), whereas the earlier trials [28,29] placed greater emphasis on three-dimensional kinematic outcomes, which may be less sensitive to the neuromuscular and pain-related changes captured by clinical measures. These differences suggest that the benefit of adding neuromuscular training to conventional physiotherapy is likely to be context- and protocol-dependent rather than uniform across all PFPS populations. Add-on designs are also sensitive to non-specific factors: Sheikhi et al., for example, reported greater improvements in pain, function and kinesiophobia when patient education was added to trunk and hip exercises [44], indicating that components delivered in addition to a standard program can influence outcomes beyond their specific content.

Pain reduction can be attributed to several factors, of which exercise therapy is one. Physiotherapy focusing on closed kinetic chain exercises has been shown to relieve pain and improve function [45]. Better temporal coordination and enhanced neuromuscular control of the quadriceps could, in theory, improve patellar alignment and reduce patellofemoral joint stress, which may in turn decrease pain [46]; however, patellar alignment, joint loading and muscle coordination were not measured in the present study, so these mechanisms remain hypothetical. Whether the additional benefit observed here is clinically important cannot be established from the present data. Baldon et al. [20] reported an MCID of 2 cm on the 10 cm VAS in women with PFPS. Against this benchmark, the median VAS score in the neuromuscular training group decreased by 2.9 cm (from 7.3 to 4.4 cm), exceeding the MCID, whereas that of the control group decreased by 1.6 cm (from 7.0 to 5.4 cm), falling short of it. Similarly, using the 10-point MCID established for the Kujala score by Crossley et al. [41], the median Kujala score in the neuromuscular training group increased by 22 points (from 55 to 77), more than twice this threshold, while that of the control group increased by 10 points (from 55 to 65), just reaching it. These comparisons provide context only: they describe within-group changes in group medians and do not show that the additional benefit of neuromuscular training exceeds the MCID for individual patients. Because individual change scores were not available, responder proportions could not be compared between groups.

Pain affects function in several ways, and function generally improves as pain decreases. Quadriceps weakness, in combination with tibial external rotation, can be addressed through quadriceps strengthening, and improved alignment may reduce pain and pressure on the patellofemoral joint [47]. Cowan et al. identified an association between pain and quadriceps inhibition in patients with PFPS [48], suggesting that a reduction in pain may improve quadriceps function. Muscle tightness is another commonly reported feature of PFPS [49], and increasing iliotibial band flexibility may reduce pressure on the lateral border of the patella and thereby improve function [6]. Adequate postural control and balance also contribute to functional movement [50]; because balance improved in both groups, functional status is likely to have improved alongside it. The greater functional improvement in the neuromuscular training group may be attributable, at least in part, to the larger reduction in pain achieved in this group, with neuromuscular training potentially promoting more effective pain-modulating motor control strategies. None of these mechanisms was measured in the present study, and the greater total exercise dose in the combined program offers an alternative explanation that does not require a mechanism specific to neuromuscular training.

Individuals with PFPS show altered movement quality during single-leg tasks, including greater dynamic knee valgus and poorer lateral step-down performance [51]. Balance test results may reflect patients’ functional status, with improvements in performance being secondary to functional gains [50]. A direct association between pain and postural instability has been reported; consequently, reducing pain and increasing muscle strength may improve balance [52]. In light of this association, the improvement in balance observed in both groups may reflect reduced nociceptive input and improved willingness to load the affected limb, although these mechanisms were not measured. The exceptionally large between-group effect sizes for dynamic balance (Cohen’s d = 4.39 for the posterolateral and d = 3.60 for the posteromedial direction) should, however, be interpreted cautiously, because effect-size estimates can be inflated when within-group variability is low. The post-treatment standard deviations for the YBT were very small, particularly in the control group (1.62–1.96 cm), relative to the difference between group means. In addition, the groups were not perfectly matched at baseline in the three reach directions, the YBT is a single-session field measure with limited measurement precision, and a ceiling effect cannot be excluded in participants approaching their maximal reach distance. Moreover, because the stance limb and selection rule underlying the reported YBT values could not be verified, it cannot be excluded that the reported values do not consistently reflect the symptomatic limb. For these reasons, the YBT results do not support any conclusion about the effect of neuromuscular training on dynamic balance, and this question requires investigation in future trials with verified, limb-specific and baseline-adjusted analyses. The supplementary change-score estimates (approximately 7.4–7.5 cm) were about 30–40% smaller than the unadjusted post-treatment differences, indicating that the unadjusted effect sizes overstate any between-group difference; however, these estimates are derived from summary statistics and do not resolve the uncertainty about the analytical definition of the YBT values.

Insufficient sleep has been linked to heightened pain sensitivity and reduced pain tolerance [16], which could impede the effective management of PFPS. Pain and sleep quality are negatively associated [17]; pain disrupts sleep continuity and shortens total sleep time, and poor sleep in turn increases pain sensitivity, creating a vicious circle that reflects the close interrelationship of the two [17,18]. A substantial body of research has identified a positive association between physical activity and sleep quality [53,54]. Kovacevic et al. reported that resistance exercise improves sleep quality and recommended combining aerobic and resistance exercise for optimal sleep quality [55], and Uchida et al. suggested that resistance exercise may improve sleep by reducing depression and anxiety and alleviating musculoskeletal pain [56]. In the present study, sleep quality was analyzed as a secondary, exploratory outcome because, unlike pain and function, no PFPS-specific mechanistic rationale for a neuromuscular-training-specific effect on sleep had been established a priori. Sleep quality improved significantly within both groups, but no significant between-group difference was observed (r = 0.03, 95% CI −0.25 to 0.31). Because this CI remains compatible with a small between-group difference in either direction, the absence of a statistically significant effect should not be read as evidence that neuromuscular training confers no additional benefit for sleep; demonstrating that would require a formal equivalence test against a pre-specified margin in a trial prospectively designed and powered for this outcome [40]. The pattern observed is consistent with sleep improving as a downstream consequence of reduced pain and increased physical activity in both groups rather than through a mechanism specific to neuromuscular training. To the best of our knowledge, this is among the first studies to examine sleep quality as an outcome of neuromuscular training in individuals with PFPS, and these exploratory findings should be confirmed in studies specifically powered for this outcome.

Although hip and knee muscle strengthening is regarded as the “gold standard” conservative treatment, with well-documented efficacy for pain, function and lower limb kinematics in PFPS [22], the effects of neuromuscular training on lower limb movement patterns and the integration of such exercises into physiotherapy programs remain under-explored [29]. The present study is one of a limited number of trials specifically designed to investigate the effect of adding neuromuscular training to conventional physiotherapy on pain, function, physical activity, balance and sleep quality in patients with PFPS.

### Strengths and Limitations

Because of the higher prevalence of PFPS in women, previous studies have predominantly included female participants; the inclusion of both men and women in this study can therefore be regarded as a strength that broadens the applicability of the findings, although the study was not designed or powered to test for sex-based differences in treatment response. Several limitations should nonetheless be acknowledged. First, the 30–60-year age range, chosen to reflect the adult population presenting to the recruitment site, differs from the adolescent and young, physically active populations most frequently emphasized in the PFPS literature, which may limit generalizability to younger or more athletic individuals. Second, the sample size calculation was based on a large anticipated effect (d = 1.04); the trial was therefore not designed to detect small between-group differences, and the non-significant findings for sleep quality and several physical activity domains should be regarded as inconclusive, since their CIs (Table 3 and Table 4) remain compatible with small but potentially relevant differences; formal equivalence testing against a pre-specified margin would be required to exclude such differences [40]. Third, lower extremity muscle strength and proprioception were not directly measured, so it is not possible to determine whether the clinical improvements are attributable to enhanced proprioceptive input, increased muscle strength or both. Fourth, the control group received active conventional physiotherapy rather than no treatment; this choice was made for ethical reasons, and the between-group differences therefore reflect the added effect of neuromuscular training over conventional therapy rather than its effect versus no intervention. Fifth, because the neuromuscular program was added to, rather than substituted for, part of the conventional program, participants in the experimental group received a greater total exercise dose, although the frequency of therapist follow-up visits was the same in both groups; the design therefore cannot distinguish the effect of the neuromuscular content from that of additional exercise exposure. Sixth, pain chronicity was defined as at least three months with no upper limit, introducing heterogeneity in symptom duration, and potential confounders such as smoking, alcohol consumption and comorbidities were not recorded, while occupation was recorded but not included in the analyses. Seventh, adherence, although complete among analyzed participants, was documented using self-completed exercise charts verified at follow-up visits rather than by objective monitoring. Eighth, because the six participants who did not perform the exercises regularly were lost to follow-up and provided no post-treatment data, the analysis was necessarily per protocol and no intention-to-treat analysis was undertaken, which may bias the estimated treatment effect in favor of the intervention; moreover, the baseline data of these participants were deleted after data collection was completed, so it could not be determined whether they differed systematically from those who completed the trial. Ninth, the analytical definition of the reported YBT values (stance limb and selection rule) could not be verified, YBT reach distances were not normalized to limb length, and the groups differed at baseline; the YBT results are therefore reported descriptively and do not support conclusions about dynamic balance. Tenth, the original paper assessment forms were securely destroyed after data entry to protect participant confidentiality, so the available participant-level dataset, compiled in December 2024, could not be verified against them; this dataset also showed inconsistencies with the reported values; it was therefore not used for re-analysis. Consequently, baseline-adjusted (ANCOVA), intention-to-treat sensitivity and responder analyses could not be performed, and the reported analyses cannot be independently re-verified. Baseline differences were addressed only through supplementary change-score analyses derived from summary statistics, whose results depend on the assumed baseline–follow-up correlation. Finally, no follow-up beyond the 12-week intervention was performed. Future studies incorporating longer-term follow-up, objective adherence monitoring, objective neuromuscular and strength measures, intention-to-treat analysis and recruitment across a broader age range are recommended.

## 5. Conclusions

In adults with PFPS, both conventional physiotherapy alone and conventional physiotherapy combined with neuromuscular training were associated with improvements in pain, function, physical activity and sleep quality over 12 weeks. Adding neuromuscular training was associated with greater short-term improvements in pain and in the primary functional outcome (Kujala score), and in walking activity, than conventional physiotherapy alone; the finding for the 30-Second Chair Stand Test was less robust. No conclusion can be drawn regarding dynamic balance, because the YBT results could not be verified and the groups differed at baseline, and the results for sleep quality and the other physical activity domains were inconclusive. Because the experimental group also received a greater total exercise dose, these findings reflect the effect of the combined program rather than a mechanism specific to neuromuscular training. Given that the reported analyses could not be independently re-verified, these results should be regarded as preliminary and require confirmation in adequately documented trials with baseline-adjusted and intention-to-treat analyses, dose-matched control programs, objective adherence monitoring and longer follow-up.

## Figures and Tables

**Table 1:** Exercise programs: (a) conventional physiotherapy program performed by both groups; (b) neuromuscular training program performed by the neuromuscular training group only: exercises, dosage and progression criteria.

Exercise	Sets × Reps/Hold	Component/Progression Criterion (Reassessed Every 3 Weeks)
**(a) Conventional physiotherapy program (both groups)**		
Walking at a comfortable pace	—	Warm-up
Active knee and hip mobility	1 × 10 each	Mobility
Straight leg raise	3 × 10–12	Strengthening
Side-lying hip abduction	3 × 10–12	Strengthening
Clamshell exercise	3 × 10–12	Strengthening
Mini squat (0–45°)	3 × 10–12	Strengthening
Glute bridge	3 × 10–12	Strengthening
Resistance-band terminal knee extension	3 × 10–12	Strengthening
Quadriceps, hamstring and gastrocnemius stretching	2 × 30 s per muscle, per side	Stretching
Slow walking	—	Cool-down
**(b) Neuromuscular training program (neuromuscular training group only)**		
Lunge to hamstring stretch	3 × 30 s hold	Increase depth of lunge and stretch range as tolerated
Side jumps	3 × 10 reps	Increase jump speed/distance as control improves
Squat	3 × 10 reps	Increase squat depth toward 90° knee flexion as tolerated
Single-leg squat	3 × 10 reps	Increase depth and reduce upper-limb support as balance improves
Forward–backward hops	3 × 10 reps	Increase hop distance/speed as control improves
Double-leg vertical jump	3 × 10 reps	Increase jump height and reduce landing contact time as tolerated
Plank	3 × 30 s hold	Build up to the full 30 s hold with correct trunk alignment as tolerated
Side plank	3 × 30 s hold	Build up to the full 30 s hold with correct trunk alignment as tolerated
Bird dog	3 × 10 reps	Increase hold time per repetition and reduce trunk compensation
Single-leg glute bridge	3 × 10 reps	Increase hold time at end-range hip extension as tolerated
Single toe raises	3 × 30 s	Increase movement speed and control within the set duration as tolerated
Monster walk	3 × 10 reps	Increase step distance and movement speed as control improves
Lateral jumps	3 × 10 reps	Increase jump distance/speed as control improves
Single-leg stand	3 × 30 s hold	Progress from bilateral hand support to no support as balance improves
Lateral lunges	3 × 30 s	Increase lunge depth and lateral step distance as tolerated

All neuromuscular training exercises were performed using body weight alone. Progression decisions were made by the supervising physiotherapist at each 3-week follow-up visit, based on demonstrated technique and symptom tolerance; sets and repetitions were held constant while task difficulty (range, speed, support or control demands) was advanced.

**Table 2:** Sociodemographic and clinical characteristics of the participants.

Variable		Neuromuscular Training Group (*n* = 25)	Control Group (*n* = 25)	*p*
Age (years)	X ± SD	44.08 ± 8.89	44.60 ± 9.28	0.841 ^t^
BMI (kg/m^2^)	X ± SD	24.38 ± 1.40	24.46 ± 1.73	0.866 ^t^
Sex	Female, *n* (%)	16 (64)	16 (64)	1.000 ^x^
	Male, *n* (%)	9 (36)	9 (36)	
Painful knee side	Right, *n* (%)	13 (52)	12 (48)	0.777 ^x^
	Left, *n* (%)	12 (48)	13 (52)	
Education	Primary school, *n* (%)	2 (8)	5 (20)	0.808 ^f^
	Middle school, *n* (%)	5 (20)	6 (24)	
	High school, *n* (%)	6 (24)	5 (20)	
	Undergraduate, *n* (%)	8 (32)	6 (24)	
	Postgraduate, *n* (%)	4 (16)	3 (12)	
Dominant side	Right, *n* (%)	19 (76)	21 (84)	0.725 ^f^
	Left, *n* (%)	6 (24)	4 (16)	

^t^ Independent-samples *t*-test; ^x^ Pearson chi-square test; ^f^ Fisher’s exact test (Fisher–Freeman–Halton test for education). X: mean; SD: standard deviation; BMI: body mass index; *n*: number of participants.

**Table 3:** Visual Analog Scale, Kujala Patellofemoral Score and International Physical Activity Questionnaire results.

Variable		Neuromuscular Training Group (*n* = 25), Median (Min–Max)	Control Group (*n* = 25), Median (Min–Max)	*p* (Between-Group, Post-Treatment)	Effect Size (Post-Treatment, Between-Group)
Visual Analog Scale	BI	7.3 (6.2–8.3)	7.0 (6.5–8.3)		
	AI	4.4 (4.2–6.2)	5.4 (4.9–6.8)	<0.001 ^m^	r = 0.72, 95% CI 0.55 to 0.83 (large)
	*p* (within-group)	<0.001 ^w^	<0.001 ^w^		
Kujala Patellofemoral Score	BI	55 (45–58)	55 (45–62)		
	AI	77 (74–84)	65 (61–78)	<0.001 ^m^	r = 0.81, 95% CI 0.69 to 0.89 (large)
	*p* (within-group)	<0.001 ^w^	<0.001 ^w^		
IPAQ–Vigorous Physical Activity	BI	160 (80–720)	480 (80–1440)		
	AI	480 (120–1800)	480 (80–1920)	0.730 ^m^	r = 0.05, 95% CI −0.23 to 0.32 (trivial)
	*p* (within-group)	<0.001 ^w^	0.007 ^w^		
IPAQ–Moderate Physical Activity	BI	180 (60–720)	480 (40–1200)		
	AI	480 (160–1200)	480 (80–1200)	0.604 ^m^	r = 0.07, 95% CI −0.21 to 0.34 (trivial)
	*p* (within-group)	<0.001 ^w^	0.001 ^w^		
IPAQ–Walking	BI	396 (99–891)	396 (66–891)		
	AI	1039.5 (594–1386)	495 (264–1039.5)	0.001 ^m^	r = 0.48, 95% CI 0.23 to 0.67 (moderate–large)
	*p* (within-group)	<0.001 ^w^	<0.001 ^w^		
IPAQ–Sitting	BI	9 (6–18)	12 (6–18)		
	AI	7.5 (6–15)	9 (6–15)	0.055 ^m^	r = 0.27, 95% CI −0.01 to 0.51 (small–moderate)
	*p* (within-group)	<0.001 ^w^	0.001 ^w^		
IPAQ–Total	BI	725 (117–2139)	808 (84–3537)		
	AI	1900.5 (903–4045.5)	1243 (392–4165.5)	0.101 ^m^	r = 0.23, 95% CI −0.05 to 0.48 (small)
	*p* (within-group)	<0.001 ^w^	<0.001 ^w^		

^w^ Wilcoxon signed-rank test; ^m^ Mann–Whitney U test. BI: before intervention; AI: after intervention; Min: minimum; Max: maximum; IPAQ: International Physical Activity Questionnaire (MET-min/week; sitting, hours/day); CI: confidence interval. Effect size r = Z/√N (N = 50) from the Mann–Whitney U test Z statistic, interpreted as trivial (<0.10), small (≥0.10), moderate (≥0.30) or large (≥0.50); 95% CIs for r were derived using the Fisher z transformation. Between-group differences for the Visual Analog Scale, Kujala score and IPAQ–walking remained significant after Bonferroni correction (adjusted α = 0.0042, 12 comparisons).

**Table 4:** 30-Second Chair Stand Test, Y Balance Test and Pittsburgh Sleep Quality Index results.

Variable		Neuromuscular Training Group (*n* = 25)	Control Group (*n* = 25)	*p* (Between-Group, Post-Treatment)	Effect Size (Post-Treatment, Between-Group)
30-Second Chair Stand Test (repetitions), X ± SD	BI	18.08 ± 2.56	18.00 ± 2.78		
	AI	22.04 ± 2.92	19.44 ± 3.12	0.004 ^t^	MD 2.60, 95% CI 0.88 to 4.32; d = 0.86, 95% CI 0.28 to 1.44 (large)
	*p* (within-group)	<0.001 ^e^	<0.001 ^e^		
YBT–Posterolateral (cm), X ± SD	BI	75.28 ± 2.75	70.40 ± 1.55		
	AI	85.40 ± 3.64	73.04 ± 1.62	<0.001 ^t^	MD 12.36 cm, 95% CI 10.74 to 13.98; d = 4.39, 95% CI 3.36 to 5.41 (large)
	*p* (within-group)	<0.001 ^e^	<0.001 ^e^		
YBT–Posteromedial (cm), X ± SD	BI	73.04 ± 2.85	69.84 ± 1.91		
	AI	82.68 ± 3.66	72.12 ± 1.96	<0.001 ^t^	MD 10.56 cm, 95% CI 8.88 to 12.24; d = 3.60, 95% CI 2.70 to 4.49 (large)
	*p* (within-group)	<0.001 ^e^	<0.001 ^e^		
YBT–Anterior (cm), Median (Min–Max)	BI	79 (70–82)	74 (70–78)		
	AI	92 (81–96)	75 (72–80)	<0.001 ^m^	r = 0.86, 95% CI 0.76 to 0.92 (large)
	*p* (within-group)	<0.001 ^w^	<0.001 ^w^		
Pittsburgh Sleep Quality Index, Median (Min–Max)	BI	16 (11–21)	16 (11–21)		
	AI	14 (10–21)	14 (10–19)	0.814 ^m^	r = 0.03, 95% CI −0.25 to 0.31 (trivial)
	*p* (within-group)	0.001 ^w^	<0.001 ^w^		

^e^ Paired-samples *t*-test; ^t^ independent-samples *t*-test; ^w^ Wilcoxon signed-rank test; ^m^ Mann–Whitney U test. X: mean; SD: standard deviation; YBT: Y Balance Test; BI: before intervention; AI: after intervention; MD: mean difference (neuromuscular training group minus control group) with 95% CI; CI: confidence interval. Cohen’s d (with 95% CI) was calculated for independent-samples *t*-test comparisons and r = Z/√N (95% CI via Fisher z transformation) for Mann–Whitney U test comparisons; d: 0.20/0.50/0.80 = small/medium/large; r: 0.10/0.30/0.50 = small/medium/large. All between-group differences in this table except the Pittsburgh Sleep Quality Index remained significant after Bonferroni correction (adjusted α = 0.0042, 12 comparisons). The Cohen’s d values for the YBT are exceptionally large and should be interpreted with caution because effect-size estimates are inflated when within-group variability is low (see Section 4). YBT values are reported descriptively; the stance limb underlying the reported values could not be verified (Section 2.6.4).

## Data Availability

A participant-level dataset compiled from the study records in December 2024 is held by the corresponding author and has been provided to the editorial office. Because it could not be verified against the original paper assessment forms, which were securely destroyed after data entry to protect participant confidentiality, and because it contains inconsistencies with the reported values, it is not publicly shared. Aggregated data supporting the reported findings are presented in the article.

## Abbreviations

The following abbreviations are used in this manuscript:

BMI	Body mass index
CI	Confidence interval
IPAQ	International Physical Activity Questionnaire
IPAQ-SF	International Physical Activity Questionnaire–Short Form
MCID	Minimal clinically important difference
MD	Mean difference
MET	Metabolic equivalent of task
PFPS	Patellofemoral pain syndrome
PSQI	Pittsburgh Sleep Quality Index
SD	Standard deviation
VAS	Visual Analog Scale
YBT	Y Balance Test

## References

[1] Alba-Martín P., Gallego-Izquierdo T., Plaza-Manzano G., Romero-Franco N., Núñez-Nagy S., Pecos-Martín D. (2015). Effectiveness of therapeutic physical exercise in the treatment of patellofemoral pain syndrome: A systematic review. J. Phys. Ther. Sci..

[2] Smith B.E., Selfe J., Thacker D., Hendrick P., Bateman M., Moffatt F., Rathleff M.S., Smith T.O., Logan P. (2018). Incidence and prevalence of patellofemoral pain: A systematic review and meta-analysis. PLoS ONE.

[3] Taunton J.E., Ryan M.B., Clement D.B., McKenzie D.C., Lloyd-Smith D.R., Zumbo B.D. (2002). A retrospective case-control analysis of 2002 running injuries. Br. J. Sports Med..

[4] Thomeé R., Renström P., Karlsson J., Grimby G. (1995). Patellofemoral pain syndrome in young women: I. A clinical analysis of alignment, pain parameters, common symptoms and functional activity level. Scand. J. Med. Sci. Sports.

[5] Neal B.S., Lack S.D., Lankhorst N.E., Raye A., Morrissey D., van Middelkoop M. (2019). Risk factors for patellofemoral pain: A systematic review and meta-analysis. Br. J. Sports Med..

[6] Piva S.R., Fitzgerald G., Wisniewski S., Delitto A. (2009). Predictors of pain and function outcome after rehabilitation in patients with patellofemoral pain syndrome. J. Rehabil. Med..

[7] Barton C.J., Webster K.E., Menz H.B. (2008). Evaluation of the scope and quality of systematic reviews on nonpharmacological conservative treatment for patellofemoral pain syndrome. J. Orthop. Sports Phys. Ther..

[8] Collado H., Fredericson M. (2010). Patellofemoral pain syndrome. Clin. Sports Med..

[9] Saad M.C., Felício L.R., Masullo C.d.L., Liporaci R.F., Bevilaqua-Grossi D. (2011). Analysis of the center of pressure displacement, ground reaction force and muscular activity during step exercises. J. Electromyogr. Kinesiol..

[10] Citaker S., Kaya D., Yuksel I., Yosmaoglu B., Nyland J., Atay O.A., Doral M.N. (2011). Static balance in patients with patellofemoral pain syndrome. Sports Health.

[11] Aminaka N., Gribble P.A. (2008). Patellar taping, patellofemoral pain syndrome, lower extremity kinematics, and dynamic postural control. J. Athl. Train..

[12] Tyler T.F., Nicholas S.J., Mullaney M.J., McHugh M.P. (2006). The role of hip muscle function in the treatment of patellofemoral pain syndrome. Am. J. Sports Med..

[13] Blond L., Hansen L. (1998). Patellofemoral pain syndrome in athletes: A 5.7-year retrospective follow-up study of 250 athletes. Acta Orthop. Belg..

[14] Reijnders L., van de Groes S.A.W. (2020). The quality of life of patients with patellofemoral pain—A systematic review. Acta Orthop. Belg..

[15] Silva D.d.O., Coura M.B., Waiteman M., Pradela J., Pazzinatto M.F., Magalhães F.H., de Azevedo F.M. (2016). Patellofemoral pain and sports practice: Reduced symptoms and higher quality of life in adolescent athletes as compared to non-athletes. Mot. Rev. Educ. Fís..

[16] Lautenbacher S., Kundermann B., Krieg J.-C. (2006). Sleep deprivation and pain perception. Sleep Med. Rev..

[17] Finan P.H., Goodin B.R., Smith M.T. (2013). The association of sleep and pain: An update and a path forward. J. Pain.

[18] Smith M.T., Haythornthwaite J.A. (2004). How do sleep disturbance and chronic pain inter-relate? Insights from the longitudinal and cognitive-behavioral clinical trials literature. Sleep Med. Rev..

[19] Fukuda T.Y., Melo W.P., Zaffalon B.M., Rossetto F.M., Magalhães E., Bryk F.F., Martin R.L. (2012). Hip posterolateral musculature strengthening in sedentary women with patellofemoral pain syndrome: A randomized controlled clinical trial with 1-year follow-up. J. Orthop. Sports Phys. Ther..

[20] Baldon R.d.M., Serrão F.V., Silva R.S., Piva S.R. (2014). Effects of functional stabilization training on pain, function, and lower extremity biomechanics in women with patellofemoral pain: A randomized clinical trial. J. Orthop. Sports Phys. Ther..

[21] Fukuda T.Y., Rossetto F.M., Magalhães E., Bryk F.F., Lucareli P.R.G., Carvalho N.A.D.A. (2010). Short-term effects of hip abductors and lateral rotators strengthening in females with patellofemoral pain syndrome: A randomized controlled clinical trial. J. Orthop. Sports Phys. Ther..

[22] Nascimento L.R., Teixeira-Salmela L.F., Souza R.B., Resende R.A. (2018). Hip and knee strengthening is more effective than knee strengthening alone for reducing pain and improving activity in individuals with patellofemoral pain: A systematic review with meta-analysis. J. Orthop. Sports Phys. Ther..

[23] Petersen W., Ellermann A., Gösele-Koppenburg A., Best R., Rembitzki I.V., Brüggemann G.P., Liebau C. (2014). Patellofemoral pain syndrome. Knee Surg. Sports Traumatol. Arthrosc..

[24] Chappell J.D., Limpisvasti O. (2008). Effect of a neuromuscular training program on the kinetics and kinematics of jumping tasks. Am. J. Sports Med..

[25] Rabelo N.D.d.A., Lima B., dos Reis A.C., Bley A.S., Yi L.C., Fukuda T.Y., Costa L.O.P., Lucareli P.R.G. (2014). Neuromuscular training and muscle strengthening in patients with patellofemoral pain syndrome: A protocol of randomized controlled trial. BMC Musculoskelet. Disord..

[26] Motealleh A., Mohamadi M., Moghadam M.B., Nejati N., Arjang N., Ebrahimi N. (2019). Effects of core neuromuscular training on pain, balance, and functional performance in women with patellofemoral pain syndrome: A clinical trial. J. Chiropr. Med..

[27] Silva N.C., Silva M.d.C., Guimarães M.G., Nascimento M.B.d.O., Felicio L.R. (2019). Effects of neuromuscular training and strengthening of trunk and lower limbs muscles in women with patellofemoral pain: A protocol of randomized controlled clinical trial, blinded. Trials.

[28] Silva N.C., Silva M.d.C., Tamburús N.Y., Guimarães M.G., Nascimento M.B.d.O., Felicio L.R. (2023). Adding neuromuscular training to a strengthening program did not produce additional improvement in clinical or kinematic outcomes in women with patellofemoral pain: A blinded randomised controlled trial. Musculoskelet. Sci. Pract..

[29] Rabelo N.D.d.A., Costa L.O.P., de Lima B.M., Dos Reis A.C., Bley A.S., Fukuda T.Y., Lucareli P.R.G. (2017). Adding motor control training to muscle strengthening did not substantially improve the effects on clinical or kinematic outcomes in women with patellofemoral pain: A randomised controlled trial. Gait Posture.

[30] Faul F., Erdfelder E., Lang A.-G., Buchner A. (2007). G*Power 3: A flexible statistical power analysis program for the social, behavioral, and biomedical sciences. Behav. Res. Methods.

[31] Cohen J. (2013). Statistical Power Analysis for the Behavioral Sciences.

[32] Hoenig J.M., Heisey D.M. (2001). The abuse of power: The pervasive fallacy of power calculations for data analysis. Am. Stat..

[33] Hawker G.A., Mian S., Kendzerska T., French M. (2011). Measures of adult pain: Visual Analog Scale for Pain (VAS Pain), Numeric Rating Scale for Pain (NRS Pain), McGill Pain Questionnaire (MPQ), Short-Form McGill Pain Questionnaire (SF-MPQ), Chronic Pain Grade Scale (CPGS), Short Form-36 Bodily Pain Scale (SF-36 BPS), and Measure of Intermittent and Constant Osteoarthritis Pain (ICOAP). Arthritis Care Res..

[34] Kujala U.M., Jaakkola L.H., Koskinen S.K., Taimela S., Hurme M., Nelimarkka O. (1993). Scoring of patellofemoral disorders. Arthroscopy.

[35] Kuru T., Dereli E.E., Yaliman A. (2010). Validity of the Turkish version of the Kujala patellofemoral score in patellofemoral pain syndrome. Acta Orthop. Traumatol. Turc..

[36] Jones C.J., Rikli R.E., Beam W.C. (1999). A 30-s chair-stand test as a measure of lower body strength in community-residing older adults. Res. Q. Exerc. Sport.

[37] Shaffer S.W., Teyhen D.S., Lorenson C.L., Warren R.L., Koreerat C.M., Straseske C.A., Childs J.D. (2013). Y-balance test: A reliability study involving multiple raters. Mil. Med..

[38] Öztürk M. (2005). Validity and Reliability of the International Physical Activity Questionnaire and Determination of Physical Activity Levels in University Students. Master’s Thesis.

[39] Ağargün M.Y., Kara H., Anlar Ö. (1996). Validity and reliability of the Pittsburgh Sleep Quality Index. Türk Psikiyatr. Derg..

[40] Lakens D. (2017). Equivalence tests: A practical primer for *t* tests, correlations, and meta-analyses. Soc. Psychol. Personal. Sci..

[41] Crossley K.M., Bennell K.L., Cowan S.M., Green S. (2004). Analysis of outcome measures for persons with patellofemoral pain: Which are reliable and valid?. Arch. Phys. Med. Rehabil..

[42] Willy R.W., Hoglund L.T., Barton C.J., Bolgla L.A., Scalzitti D.A., Logerstedt D.S., Lynch A.D., Snyder-Mackler L., McDonough C.M., Altman R. (2019). Patellofemoral pain: Clinical practice guidelines linked to the International Classification of Functioning, Disability and Health from the Academy of Orthopaedic Physical Therapy of the American Physical Therapy Association. J. Orthop. Sports Phys. Ther..

[43] Prentice W.E., Voight M.L. (2003). Técnicas em Reabilitação Musculoesquelética.

[44] Sheikhi B., Rabiei P., Letafatkar A., Rossettini G. (2024). Is adding education to trunk and hip exercises beneficial for patellofemoral pain? A randomized controlled trial. Arch. Phys. Med. Rehabil..

[45] Balci P., Tunay V.B., Baltaci G., Atay A.O. (2009). The effects of two different closed kinetic chain exercises on muscle strength and proprioception in patients with patellofemoral pain syndrome. Acta Orthop. Traumatol. Turc..

[46] Boling M.C., Bolgla L.A., Mattacola C.G., Uhl T.L., Hosey R.G. (2006). Outcomes of a weight-bearing rehabilitation program for patients diagnosed with patellofemoral pain syndrome. Arch. Phys. Med. Rehabil..

[47] Wünschel M., Leichtle U., Obloh C., Wülker N., Müller O. (2011). The effect of different quadriceps loading patterns on tibiofemoral joint kinematics and patellofemoral contact pressure during simulated partial weight-bearing knee flexion. Knee Surg. Sports Traumatol. Arthrosc..

[48] Cowan S.M., Bennell K.L., Hodges P.W., Crossley K.M., McConnell J. (2001). Delayed onset of electromyographic activity of vastus medialis obliquus relative to vastus lateralis in subjects with patellofemoral pain syndrome. Arch. Phys. Med. Rehabil..

[49] White L.C., Dolphin P., Dixon J. (2009). Hamstring length in patellofemoral pain syndrome. Physiotherapy.

[50] Kisner C., Colby L.A., Borstad J. (2017). Therapeutic Exercise: Foundations and Techniques.

[51] Emamvirdi M., Hosseinzadeh M., Letafatkar A., Thomas A.C., Dos’Santos T., Smania N., Rossettini G. (2023). Comparing kinematic asymmetry and lateral step-down test scores in healthy, chronic ankle instability, and patellofemoral pain syndrome female basketball players: A cross-sectional study. Sci. Rep..

[52] Hunt M.A., McManus F.J., Hinman R.S., Bennell K.L. (2010). Predictors of single-leg standing balance in individuals with medial knee osteoarthritis. Arthritis Care Res..

[53] Driver H.S., Taylor S.R. (2000). Exercise and sleep. Sleep Med. Rev..

[54] Kelley G.A., Kelley K.S. (2017). Exercise and sleep: A systematic review of previous meta-analyses. J. Evid. Based Med..

[55] Kovacevic A., Mavros Y., Heisz J.J., Singh M.A.F. (2018). The effect of resistance exercise on sleep: A systematic review of randomized controlled trials. Sleep Med. Rev..

[56] Uchida S., Shioda K., Morita Y., Kubota C., Ganeko M., Takeda N. (2012). Exercise effects on sleep physiology. Front. Neurol..

